# A ‘suicide’ CRISPR-Cas9 system to promote gene deletion and restoration by electroporation in *Cryptococcus neoformans*

**DOI:** 10.1038/srep31145

**Published:** 2016-08-09

**Authors:** Yu Wang, Dongsheng Wei, Xiangyang Zhu, Jiao Pan, Ping Zhang, Liang Huo, Xudong Zhu

**Affiliations:** 1 National Key Program of Microbiology and Department of Microbiology, College of Life Sciences, Nankai University (DMNU), Tianjin 300071, China; 2 Beijing Key Laboratory of Genetic Engineering Drug and Biotechnology, Institute of Biochemistry and Biotechnology, College of Life Sciences, Beijing Normal University, Beijing 100875, China

## Abstract

Loss-of-function mutagenesis is an important tool used to characterize gene functions, and the CRISPR-Cas9 system is a powerful method for performing targeted mutagenesis in organisms that present low recombination frequencies, such as the serotype D strains of Cryptococcus neoformans. However, when the CRISPR-Cas9 system persists in the host cells, off-target effects and Cas9 cytotoxicity may occur, which might block subsequent genetic manipulation. Here, we report a method of spontaneously eliminating the CRISPR-Cas9 system without impairing its robust editing function. We successfully expressed single guide RNA under the driver of an endogenous U6 promoter and the human codon-optimized Cas9 endonuclease with an *ACT1* promoter. This system can effectively generate an indel mutation and efficiently perform targeted gene disruption via homology-directed repair by electroporation in yeast. We then demonstrated the spontaneous elimination of the system via a cis arrangement of the CRISPR-Cas9 expression cassettes to the recombination construct. After a system-mediated double crossover, the CRISPR-Cas9 cassettes were cleaved and degraded, which was validated by Southern blotting. This ‘suicide’ CRISPR-Cas9 system enables the validation of gene functions by subsequent complementation and has the potential to minimize off-target effects. Thus, this technique has the potential for use in functional genomics studies of *C. neoformans*.

As an adaptive immune mechanism in prokaryotes, clustered regularly interspaced short palindromic repeats (CRISPR) and CRISPR-associated (CRISPER-Cas) systems confer host defence against bacteriophages and other mobile elements and vectors^1^,^2^,^3^. The harnessed CRISPR-Cas9 systems have been acclaimed for their efficacy in genome editing in organisms that are particularly refractory to genomic manipulation^4^,^5^. To construct the system, two essential components are required: a single chimeric guide RNA (gRNA) component created by fusing an approximately 20 nt target-sequence RNA (crRNA) to a tailored bacterial tracrRNA and a protein component of the bacterial Cas9 endonuclease^6^,^7^,^8^. The system, which is in DNA form by archetype, is usually transformed into the host cells for functional expression. The expression cassettes for the production of gRNA and Cas9 nuclease will largely persist in the host as ectopic integrated copies or as free replicable DNA molecules (minichromosomes) even after the editing process is complete. Thus, inherent drawbacks occur with this approach, such as the potential cytotoxicity of Cas9 endonuclease and off-target mutations that accumulate with cell proliferation^5^,^9^. Off-target editing by the CRISPR-Cas9 system that triggers undesirable genetic alterations commonly occurs^10^,^11^, and CRISPR-Cas9 is cytotoxic in certain organisms, including *Saccharomyces cerevisiae* and *Schizosaccharomyces pombe*^12^,^13^,^14^. Another pragmatic problem encountered in our study is that the persistence of the CRISPR-Cas9 system in the cells precludes the restoration of the mutated gene in *C. neoformans* because the system also targets the complement gene that contains the same target sequence of gRNA. This general limitation represents a significant issue that must be addressed in practical applications of the editing system because the *in vivo* presence of CRISPR-Cas9 expression cassettes is not appropriate for a number of applications (*e.g*., biomedical or agricultural). Even for bench studies of microbes, the residual CRISPR-Cas9 DNA decreases the favourability of the system as a tool for genome manipulation.

The basidiomycetous yeast *C. neoformans* has been well studied as a model of AIDS-associated mycosis and other basic biological issues^15^,^16^ and presents a low homologous recombination frequency, especially for the serotype D strains. Two different transformation systems, biolistic transformation and electroporation, have been developed to disrupt a number of genes by homologous recombination. However, the frequency of homologous recombination achieved by these approaches differs dramatically between A and D serotype strains^17^. Biolistic transformation is a costly procedure that can achieve gene disruption by homologous recombination in serotype A strains at frequencies between 2 and 50% and congenic serotype D strain series at frequencies between 1 and 4%. Furthermore, transformation by electroporation in serotype D strains results in homologous recombinations at frequencies of 1/1000 to 1/100,000^18^,^19^,^20^,^21^. This result is partially caused by the preferential maintenance of transformed plasmids as stable extrachromosomal molecules through the addition of telemetric sequences at the ends^22^. Other genomic editing techniques, such as zinc-finger nucleases or TAL effector nuclease (TALENS), have not been reported in this fungus. Thus, establishing a functional CRISPR-Cas9 system to perform targeted gene deletion and gene complementation for virulence studies in yeast is of significance. Here, we report the establishment of a practical and feasible CRISPR-Cas9 system for gene targeting and restoration in *C. neoformans*.

## Results

### Construction of a CRISPR-Cas9 system for genome editing in *C. neoformans*

**Figure 1:**
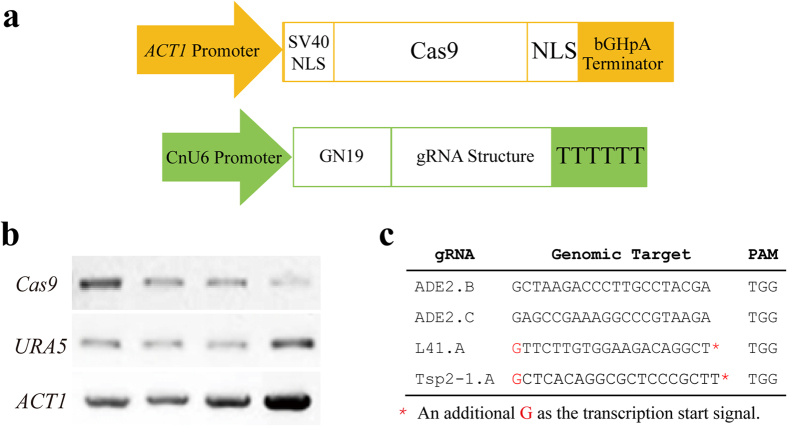
Construction of the CRISPR-Cas9 expression cassettes for genome editing in *C. neoformans*. (**a**) Schematic of the Cas9 and gRNA expression cassettes. Upper panel: the human codon-optimized Cas9 was placed behind an 800 bp promoter sequence of the cryptococcal *ACT1* gene (designated as Pact in the text). Cas9 CDS was flanked by the SV40 NLS at the N-terminus and the nucleoplasmin NLS at the C- terminus, and bGHpA serves as the terminator of the cassette. Bottom panel: gDNA expression cassette consisting of a targeting sequence, GN_19_, which was fused by overlap PCR to the tracrRNA scaffold under the CnU6 promoter and included a termination signal of 6 Ts. (**b**) Detection of Cas9 and the marker *URA5* mRNA by reverse transcription PCR to verify transcription in the transformants using *ACT1* mRNA as the internal control. Full-length gels are presented in Supplementary Fig. S1b. The gels were run under the same experimental conditions. (**c**) Target and PAM sequences for editing. Two target sites, ADE2.B and ADE2.C, were chosen for *ADE2*, L41.A was selected for *L41* and Tsp2-1.A was selected for *Tsp2-1*. L41.A is located on the antisense strand of *L41*.

We initially harnessed the type II CRISPR-Cas9 system to function in *C. neoformans* by constructing two cassettes that produce gRNA and Cas9 nuclease in the yeast (Fig. 1a). A human codon-optimized version of Cas9 is driven by a promoter of the cryptococcal actin-encoding gene *ACT1* and ends with a bGHpA terminator^6^,^8^. Two nuclear localization signal (NLS) sequences, SV40 NLS and nucleoplasmin NLS, were first fused to the N- and the C-terminus of Cas9, respectively (Fig. 1a, upper panel). Because the RNA polymerase III promoter of the U6 small RNA gene is a strong driver of gRNA production, it was used for the transcription of gRNA in yeast. We tested the human U6 promoter^6^ and the baker’s yeast SNR52 promoter^12^, although we did not achieve success. We then searched for native U6 genes in the genome of *C. neoformans,* which are not well defined. After testing several U6-like promoters, we obtained a 273 nt promoter sequence that functioned efficiently from one of the U6 candidates and designated it as CnU6 (Table S1). The DNA sequence that produced the single gRNA in the cassette consisted of two parts: a 20 nt target sequence at the 5′-end, GN_19_ (N for any base), and the subsequent DNA encoding the tracrRNA at the 3′-end (Fig. 1a, bottom panel)^6^,^7^,^8^. This entire DNA sequence that corresponds to the single gRNA transcript is hereafter denoted as gDNA when referring to the DNA cassette. The gDNA was then placed under the control of the CnU6 promoter and tailed with a 6-T terminator. The Cas9 and gDNA cassettes were loaded to the proper vectors as indicated in each case; *e.g*., pBS-URA5 (*URA5* is the marker for transformation) (Fig. S1a). The mRNAs of Cas9 and *URA5* were detected by reverse transcription PCR in the recipient strain 4500FOA (*ura5*^*−*^) (Fig. 1b; Fig. S1b).

### Targeted mutagenesis with the CRISPR-Cas9 system

**Figure 2:**
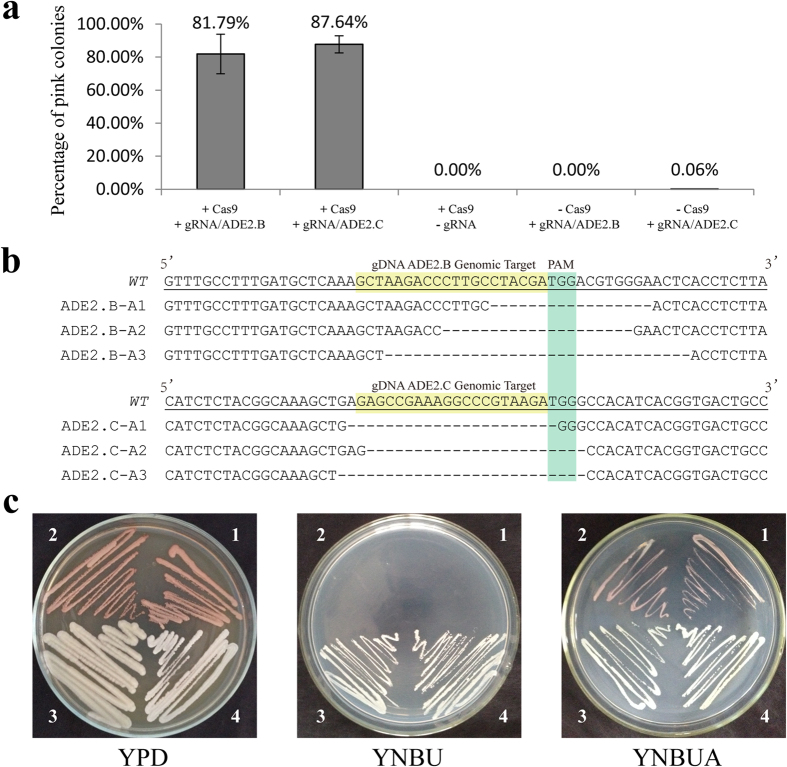
Mutagenesis with the CRISPR-Cas9 system. (**a**) Percentage of pink colonies on the YNBA plates. Only the *URA5* positive transformants containing both Cas9 and gRNA formed a large proportion of pink colonies. The error bars represent the standard deviation among three independent experiments. (**b**) Indel mutations at the target sites of *ADE2* were confirmed by sequencing of the PCR products. WT: the wild-type *ADE2*; A1-3: transformants. Target sequences and PAM are highlighted. (**c**) Phenotype of the mutants of *ade2Δ*. All of the strains grew properly on the YPD plate (left). Mutants (No. 1 and No. 2) failed to grow on the minimal medium YNB plus 40 mg/L uracil (YNBU, middle), whereas the control strains (No. 3 and No. 4) grew. The right plate is YNB supplemented with 40 mg/L uracil and 20 mg/L adenine (YNBUA) for the growth of the mutants. The mutants turned to red colonies because of mutated *ADE2* that accumulates a pigment. All plates were incubated at 30 °C for 4 days. No. 1. ADE2.B-A1, No. 2. ADE2.C-A1, No. 3. JEC21 (WT), and No. 4. 4500FOA (the recipient strain).

To examine the functionality of this system, we first targeted the *ADE2* gene encoding a phosphoribosylaminoimidazole carboxylase in the biosynthetic pathway of adenine. A loss-of-function mutation in *ADE2* results in an adenine auxotroph that forms pink colonies on culture plates that contain a low level of adenine, thereby enabling a visual evaluation of the action of CRISPR-Cas9^23^. Two target sequences near the 5′ region of the *ADE2* coding sequence (CDS), ADE2.B and ADE2.C (20 nt), were chosen (Fig. 1c). Upon transforming the linearized vectors carrying both the Cas9 and the gDNA cassettes into 4500FOA, a large proportion of *URA5*-positive transformants formed pink colonies on the YNBA plates, indicating that successful targeting occurred at the *ADE2* locus by the system (Fig. S1c). Approximately 82% and 88% of the transformants acquired from the targeted transformation of ADE2.B and ADE2.C turned pink, respectively, whereas the transformants that only contained the Cas9 or gDNA component did not produce pink colonies (Fig. 2a). We then randomly selected three pink colonies from each transformation and used PCR followed by sequencing to examine the changes in the ADE2.B and ADE2.C loci in the transformants. This analysis revealed various indel mutations at the loci (Fig. 2b). The mutations may have resulted from a mechanism of non-homologous end joining (NHEJ) initiated by the CRISPR-Cas9 system^6^,^8^. NHEJ-equivalent machinery has been described in *C. neoformans*^24^. The mutant strains failed to grow on YNBU, although they manifested adenine deficiency on YNBU supplemented with adenine (Fig. 2c). These results demonstrate that the engineered CRISPR-Cas9 system can perform high-efficiency genome editing in *C. neoformans*. Thus, the CRISPR-Cas9 system has the potential for use as a tool for functional genomics studies of *C. neoformans*, which is important because of the difficulty of gene targeting in this organism.

### CRISPR-Cas9 stimulates homologous recombination with donor DNA

**Figure 3:**
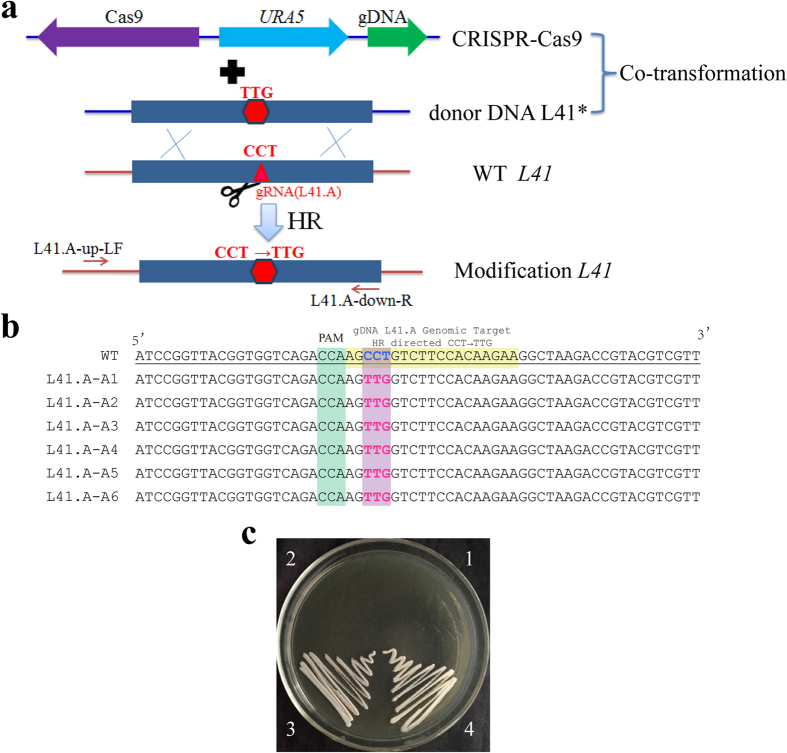
CRIPSR-Cas9-mediated modification of *L41* via homologous recombination. (**a**) Diagram of the donor DNA (modified L41*) and CRISPR-Cas9 cassettes (*URA5* as marker) for the modification of the 56^th^ codon of *L41*. The donor DNA contained a leucine codon (TTG) in place of the proline CCT in the wild-type *L41*. The gRNA (L41.A) target locus is indicated by a red triangle, where a DSB was generated by Cas9 to promote HR via double cross-over between the donor DNA L41* and the genome copy of *L41*. The primers used for PCR verification and sequencing are located within the arrows. (**b**) HR was confirmed by sequencing the PCR products from six selected mutants. The top row represents the wild-type *L41*. The PAM sequence is shown in green. The modified codon is in purple. The gRNA target sequence is indicated by yellow. (**c**) Two purified transformants, L41.A-A1 and L41.A-A2, were resistant to 20 mg/L cycloheximide on the YPD plate, whereas JEC21 and 4500FOA failed to grow. No. 1. JEC21, No. 2. 4500FOA, No. 3. L41.A-A1, and No. 4. L41.A-A2.

We subsequently tested whether the CRISPR-Cas9 system can be used to modify a specific genome locus in yeast, which likely occurs via a homology-directed repair mechanism guided by a template DNA. The target, the *L41* gene, encodes the ribosomal large subunit protein L41. This protein is the action target of the drug cycloheximide, which inhibits the growth of *C. neoformans*. Substitution of the 56^th^ amino acid (proline) by leucine confers resistance to cycloheximide^25^. We created a mutated copy of L41* that is 1000 bp in length and contains a leucine codon (TTG) in place of the proline CCT as the donor DNA (Fig. 3a). The gRNA for the Cas9 target, L41.A, striding CCT codon was accordingly designed (Fig. 1c). The CRISPR-Cas9 cassettes and the L41* donor DNA were co-transformed by electroporation into 4500FOA on cycloheximide-containing plates. The Cas9 cassette alone and the L41* donor DNA were co-transformed as a control. Dozens of transformants grew on the cycloheximide-containing plates for the CRISPR-Cas9 cassettes and the L41* donor DNA co-transformation experiment, whereas co-transformation of the Cas9 cassette alone and the L41* donor DNA did not yield transformants on cycloheximide-containing plates. The experiment was replicated three times. Six colonies grown on cycloheximide-containing plates were randomly selected to observe the CCT site by PCR sequencing with a pair of primers, L41-up-LF/L41-down-R, which are located outside of the donor DNA (Fig. 3a). Sequencing of the PCR products confirmed that all six transformants acquired a TTG codon in the native locus of CCT (Fig. 3b). Phenotypically, the transformants were resistant to antibiotics (Fig. 3c). Because there are six canonical genetic codons for leucine (TTA, TTG, CTT, CTC, CTA and CTG), the frequency of spontaneous mutation from CCT to TTG is low. This result confirms that the CRISPR-Cas9 system can improve homologous recombination frequencies with donor DNA for the *C. neoformans* serotype D strain.

### CRISPR-Cas9-mediated targeted gene deletion via homologous recombination

**Figure 4:**
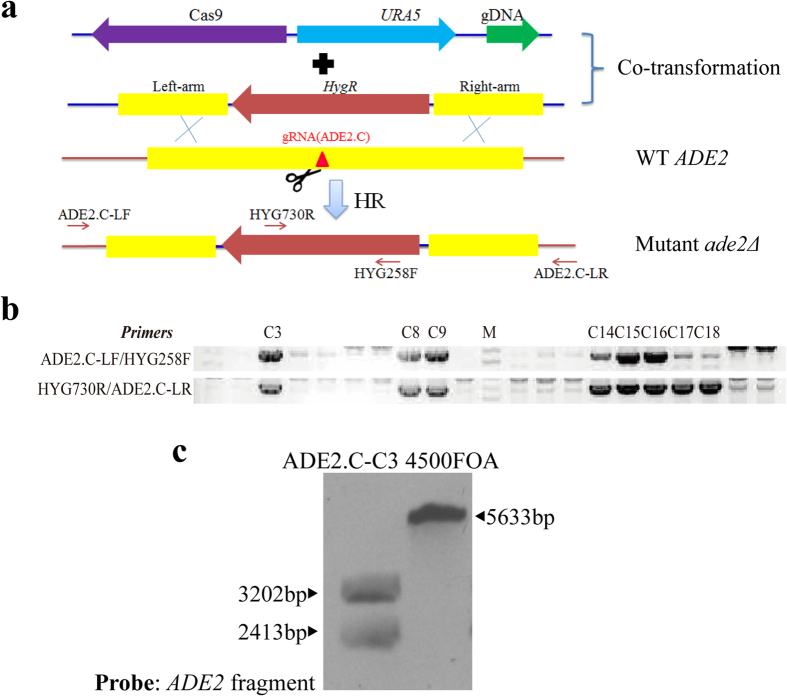
CRISPR-Cas9-mediated targeted gene deletion/replacement via homologous recombination. (**a**) Diagram of the targeted deletion of *ADE2* via CRISPR-Cas9-mediated HR. The gRNA target locus is shown as a red triangle, where DSB was supposed to be generated by Cas9. The primers used for PCR verification are indicated by the arrows. (**b**) PCR screening for deletion mutants produced by CRISPR-Cas9-mediated HR. Eight of the twenty selected transformants acquired two anticipated PCR bands in C3 to C18. The primers used for amplification are indicated on the left of the gel photographs. Full-length gels are presented in Supplementary Fig. S2. The gels were run under the same experimental conditions. (**c**) Southern blotting to confirm that *ADE2* was disrupted as speculated. A randomly selected mutant strain, ADE2.C-C3, and the control 4500FOA were analysed. The membrane was probed with a fragment of *ADE2* that was amplified by PCR with the primers ADE2 Probe-F/ADE2 Probe-R. Genomic DNA was digested with *Bam*HI and *Cla*I. The control 4500FOA had a wild-type *ADE2* band (5633 bp), and the mutant strain ADE2.C-C3 had two bands (3202 bp and 2413 bp). Full-length blots are presented in Supplementary Fig. S3b. The blots were run under the same experimental conditions.

Gene deletion through homologous recombination is routinely used in the study of gene function in *C. neoformans*. Because of the low rate of HR (10^−3^ to 10^−5^ by electroporation in JEC21), this step is laborious and costly^17^, although attempts to improve the rate of HR during gene deletion in *C. neoformans* are common^26^. Because the CRISPR-Cas9 system was demonstrated to stimulate HR, we decided to investigate whether it increased the efficiency of gene deletion/knockout in *C. neoformans* by constructing a deletion construct of *ADE2* (Fig. 4a). Two homologous arms of *ADE2*, which were approximately 1.0 kb each and 50 bp apart, were obtained from the flanking sequences of the target locus ADE2.C (gRNA) and ligated to a hygromycin-resistant gene *HygR*. The construct was then co-electroporated with CRISPR/ADE2.C cassettes into 4500FOA. Twenty pink transformants were analysed by PCR amplification and sequenced with the two pairs of primers shown in Fig. 4a. PCR amplification and its subsequent sequencing suggested that 8 of 20 transformants acquired the anticipated fragments (Figs 4b and S2), which suggested that correct gene deletion occurred at the target locus in these transformants. Southern blotting for transformant C3 confirmed the desired disruption of *ADE2* by the marker *HygR* via double crossover at the target site, which was expected (Figs 4c and S3). As a control, the deletion construct of *HygR* flanking the *ADE2* homologous arms was transformed alone into 4500FOA; however, none of the transformants were pink. Twenty transformants were also randomly selected to screen for homologous recombination by PCR. However, transformants with a disrupted *ADE2* were not detected (0/20). These results clearly demonstrate that the CRISPR-Cas9 system can dramatically increase the rate of gene deletion via a HR mechanism in *C. neoformans*.

### Spontaneous elimination of the CRISPR-Cas9 system with editing

Gene complementation is used to investigate the roles of genes in pathogenesis in *C. neoformans* to ensure that a direct relationship occurs between phenotypic consequences and the artificial mutation^27^,^28^. However, if a wild-type copy of the target gene is re-introduced into the mutant, it is modified by the persistent CRISPR-Cas9 in the yeast. To develop a CRISPR-Cas9 system that is suitable for gene complementation, we eliminated the gDNA and Cas9 expression cassettes while preserving the editing action. We designed a ‘suicide’ CRISPR-Cas9 system by a cis arrangement of gDNA and Cas9 cassettes to one side of the homologous arms of the deletion construct. The rationale behind this design relies on the observation that the flanking DNAs of the homologous arms are resolved and degraded by double crossover in homologous recombination^29^.

**Figure 5:**
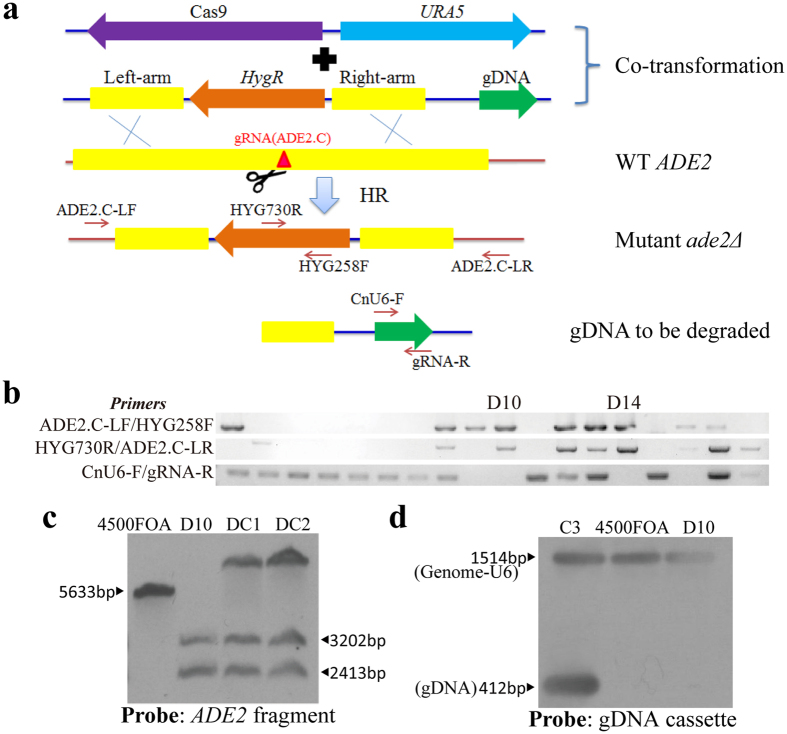
Spontaneous elimination of gDNA associated with CRISPR-Cas9-mediated gene targeting. (**a**) Structure of the editing construct for the elimination of gDNA. gDNA was cis fused to the right homologous arm in the deletion construct. The gRNA (ADE2.C) locus is indicated by the red triangle, where a DSB was generated by Cas9 to promote HR via double cross-over. The resolved DNA fragment containing gDNA is also indicated (bottom row). The primers used for PCR verification are indicated by the arrows. (**b**) Three groups of PCR screening for disrupted *ADE2* mutants that lost gDNA. Six of the transformants underwent the anticipated double crossover (the upper two rows). Two of the six, D10 and D14, lost the gDNA band (the bottom row). The primers are shown on the left. The gels were run under the same experimental conditions, and full-length gels are presented in Supplementary Fig. S4. (**c**) Verification of disruption of *ADE2* by Southern blotting in the mutant D10 (ADE2.C-D10) and two *ADE2* complement strains, DC1 and DC2, probed by the same *ADE2* fragment as in Fig. 4c. Genomic DNA was digested with *Bam*HI and *Cla*I. The control 4500FOA had a wild-type *ADE2* band (5633 bp), and the mutant strain D10 had two bands (3202 bp and 2413 bp), whereas the complements contained an additional copy of the *ADE2* fragment. (**d**) Southern blotting to demonstrate the elimination of gDNA in the *ade2Δ* mutant D10. D10 and the control 4500FOA did not contain the 412 bp gDNA band, whereas gDNA was detected in the mutant C3 that was obtained by non-suicide CRISPR-Cas9 (Fig. 4a,b). The 1541 bp wild-type U6 band was confirmed in all three strains. The blots were run under the same experimental conditions, and full-length blots are presented in Supplementary Figs S3b and S5b.

As an initial test, a gDNA cassette carrying the *ADE2* target site ADE2.C was designed. The gDNA was placed to the side of the homologous fragment in the deletion construct of *ADE2* and then co-transformed with the Cas9 cassette on a separate vector (Fig. 5a). Several pink colonies grew on the screening plates for the co-transformation experiment, whereas the transformation of only the *ADE2* deletion construct as a control did not yield pink transformants. Eighteen pink transformants were randomly selected for analysis by PCR. Three groups of PCR amplification revealed that six of the transformants had undergone the anticipated homologous recombination via double crossover. Two of the six, D10 and D14, lost the gDNA band, indicating that the gDNA fragment was degraded (Figs 5b and S4). We further verified the disruption of *ADE2* by Southern blotting (Figs 5c and S3) and the elimination of gDNA in D10 (Figs 5d and S5a,b). The results of a phenotypic assay were also consistent with the disruption of *ADE2* (Fig. S5c). These results suggest that gDNA can be eliminated without eliminating its role in the CRISPR-Cas9 system.

**Figure 6:**
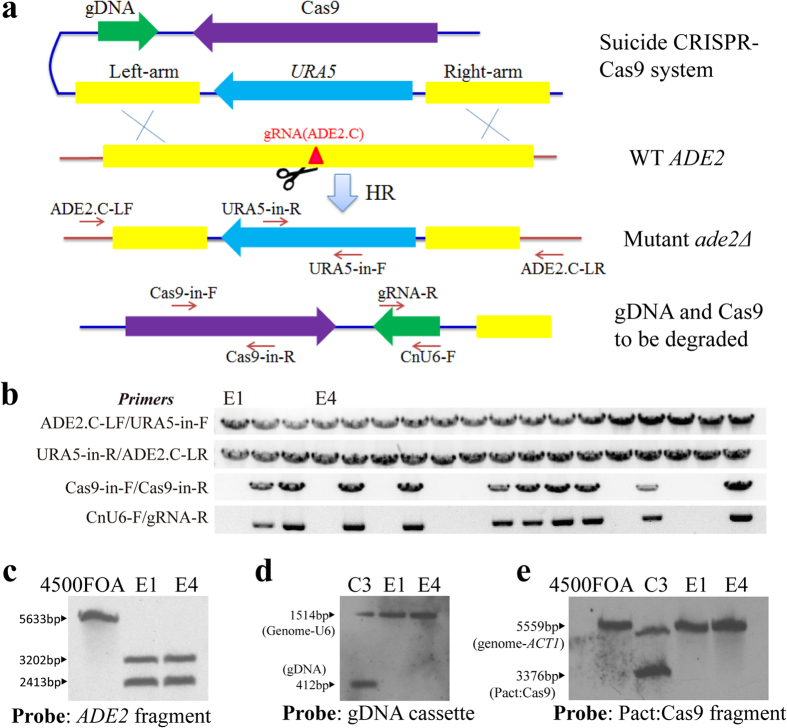
Demonstration of spontaneous elimination of both Cas9 and gDNA expression cassettes upon CRISPR-Cas9-mediated *ADE2* gene disruption. (**a**) Construction of a suicide CRISPR-Cas9 cassette. The target gRNA (ADE2.C) is indicated by a red triangle, where a DSB was generated by Cas9 to promote HR via double cross-over between *ADE2* homologous arms on the cassette and the genome copy of *ADE2*. The resolved DNA fragment containing Cas9 and gDNA is also indicated (bottom row). The primers used for PCR verification are indicated by the arrows. (**b**) Confirmation of *ADE2* disruption with PCR amplification in eighteen pink mutants. Four groups of PCR amplifications were performed for confirmation of the deletion of *ADE2* (the upper two rows) and the loss of Cas9 (the third row from the top) and gDNA (the bottom row). Primers are indicated on the left side. (**c–e**) Southern blot confirmation of *ADE2* disruption and the elimination of CRISPR-Cas9 in the *ade2Δ* mutants E1 and E4. The left panel (**c**) shows the disruption of *ADE2* probed by the same *ADE2* fragment used in Fig. 4c. Control: 4500FOA had a wild-type *ADE2* band (5633 bp), and the mutants had two bands. The middle (**d**) and the right (**e**) panels show the loss of the gDNA and Cas9 cassettes in the two mutants. Control: C3, which bears gDNA and Cas9 cassettes. C3. ADE2.C-C3, E1. ADE2.C-E1, and E4. ADE2.C-E4. Full-length gels and blots are presented in Supplementary Figs S6 and S7b-d. The gels and blots were run under the same experimental conditions.

We next tested whether a larger fragment could be eliminated because a larger DNA fragment might be more stable in *C. neoformans*^22^. The fragment included the expression cassettes of gDNA and Cas9 and was over 5.0 kb in length. The gDNA and Cas9 cassettes were similarly placed to the left arm of the recombination construct in a single vector (Fig. 6a). The linearized vector was introduced into 4500FOA. A large number of transformants (estimated at more than 90%) turned pink on the YNBA plates, whereas few pink transformants were observed in colonies that were electroporated with only the *ADE2* deletion construct as a control. Upon analysing eighteen pink colonies by PCR, we determined that all of them had undergone homologous recombination as expected (Fig. 6b, the upper two rows; Fig. S6a,b). Furthermore, eight of these colonies (8/18) had lost the Cas9 and gDNA cassettes (Fig. 6b, the bottom two rows; Fig. S6c,d), whereas the other ten transformants still harboured the cassettes. Two of the transformants, E1 and E4, were selected for Southern blot analysis, which is shown in Fig. 6c–e and Supplementary Figs S3a, S5a and S7. The results confirmed the disruption of *ADE2* and the loss of Cas9 and gDNA, whereas the control transformant C3 presented the gDNA and Cas9 cassettes. Interestingly, a high efficiency of homologous disruption was obtained in this experiment, which may have been caused by the placement of the CRISPR-Cas9 cassettes and donor DNA on a single vector in this transformation. Taken together, gDNA and Cas9 can be readily eliminated, regardless of size, and their function can be properly maintained for editing.

**Figure 7:**
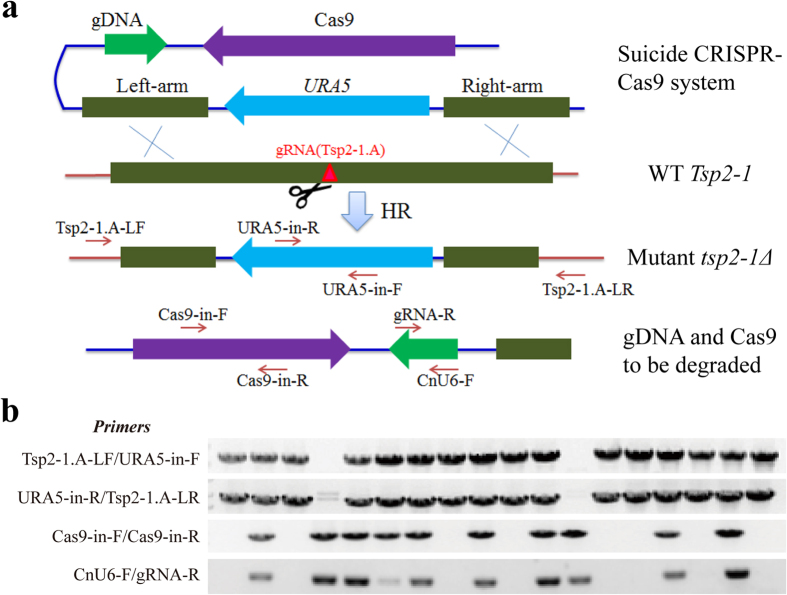
Spontaneous elimination of both Cas9 and gDNA cassettes upon CRISPR-Cas9-mediated *Tsp2-1* gene disruption. (**a**) Schematic diagram showing the construction of a disruption construct of *Tsp2-1* and the elimination of Cas9 and gDNA cassettes. The target locus (gRNA Tsp2-1.A) is indicated by a red triangle, where a DSB was supposedly created by Cas9 activity to promote HR via double crossover between the *Tsp2-1* homologous arms and the genomic copy of *Tsp2-1*. The excised DNA fragment containing Cas9 and gDNA cassettes is also indicated. The primers used for PCR verification are indicated by the arrows. (**b**) Confirmation of *Tsp2-1* disruption by PCR amplification in eighteen *tsp2-1Δ* mutants. Four groups of PCRs were conducted for confirmation of the deletion of *Tsp2-1* (the upper two rows), and the loss of Cas9 (the third row from the top) and gDNA (the bottom). Primers are indicated on the left side. Full-length gels are presented in Supplementary Fig. S8. The gels were run under the same experimental conditions.

To test whether other genes could be similarly disrupted and the CRISPR-Cas9 system could be spontaneously eliminated, we targeted another gene, *Tsp2-1,* which encodes a tetra-transmembrane protein involved in glucose signalling in *C. neoformans*^27^ (Fig. 7a). *Tsp2-1* was disrupted in sixteen out of the eighteen randomly selected transformants in which homologous recombination occurred as expected. At a similar rate, eight of the transformants had lost the Cas9 and gDNA cassettes (Figs 7b and S8). As a control, only the *Tsp2-1* deletion construct was electroporated into 4500FOA. Eighteen control transformants were randomly selected to detect the disruption of *Tsp2-1* by two groups of PCR. However, none of the predicted bands were observed in the eighteen transformants.

### Elimination of CRISPR-Cas9 allows subsequent complementation

**Figure 8:**
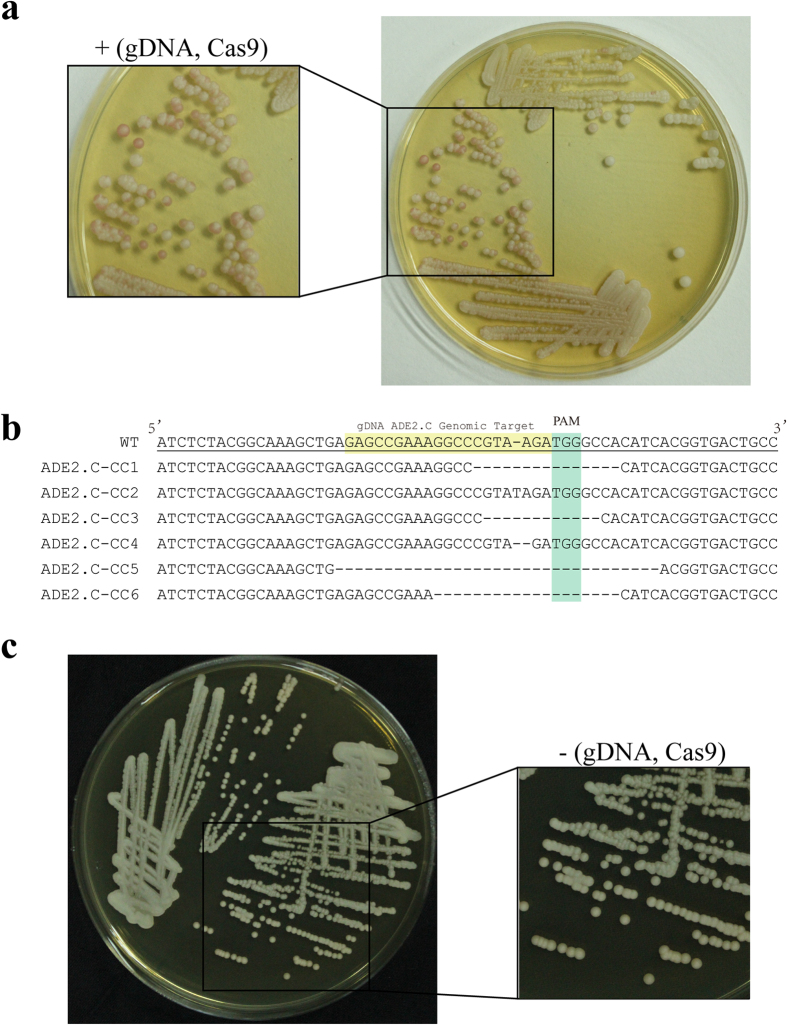
Elimination of the CRISPR-Cas9 system enables subsequent gene restoration. (**a**) Persistent CRISPR-Cas9 *in vivo* prevents the complementation of *ADE2* in ADE2.C-C3. (**b**) Sequencing for the ADE2.C locus in the red colonies (Fig. 8a) to show *de novo* mutations in the complements generated from the ADE2.C-C3 strain. (**c**) Elimination of CRISPR-Cas9 enables the successful *ADE2* complementation in E1 and E4.

To show that the loss of CRISPR-Cas9 is necessary for later gene complementation, we transformed a wild-type copy of *ADE2* back into E1 and E4 with C3 as the control containing both the gDNA and Cas9 cassettes. When a purified adenine prototrophic transformant from C3 was spread on the YPD plates, pink colonies continued to emerge with mitotic growth (Fig. 8a). Sequencing the locus ADE2.C of the reintroduced *ADE2* gene in six random pink colonies verified that new indel mutations distinct from the disruption in C3 were newly generated by the remaining CRISPR-Cas9 system (Fig. 8b). All of the complements from E1 and E4 remained in light colonies, and few turned pink on the YPD plates after elongated incubation (Fig. 8c). This result suggests that the persistence of the CRISPR-Cas9 system prevents subsequent genomic manipulation, such as gene restoration or correction. Thus, the elimination of the system by our strategy is feasible for gene complementation assays.

## Discussion

In this work, we present a bacterial-type II CRISPR-Cas9 system as a novel genome mutagenesis technique in the serotype D strain of the basidiomycetous pathogenic yeast *C. neoformans*, which presents a low frequency of homologous recombination for gene disruption^17^,^22^. By utilizing an endogenous U6 promoter, we expressed the target RNA and the bacterial tracrRNA scaffold, which were fused to form a single gRNA transcript. Our data showed that the human codon-optimized Cas9 endonuclease functioned properly in this yeast when driven by the native *ACT1* promoter. Additionally, the nuclear translocation signal peptides suitable for human cells containing Cas9 functioned efficiently in *C. neoformans*. We successfully performed several editing tasks with this system in this fungus, including the creation of indel mutations at the target loci and the modification of the gene encoding the ribosomal protein L41. Most importantly, we conducted a targeted gene deletion of *ADE2* and *Tsp2-1* at a high frequency (over 80% of all transformants). Although the indel efficiencies and homologous recombination rates for different target sites differed, the efficiency was still high (approximately 40–90% for indel and 20–90% for HR as assessed by summarizing all of the genome targeting efficiencies and homologous recombination rates). In addition, we found that optimizing the target sites by sgRNAcas9 software and shortening the distance (<30 nt) between the Cas9 cut site and the proximal ends of the homologous arms could increase the frequencies of indel- and homology-directed repair. We expect that the CRISPR-Cas9 editing system could become a valuable tool for facilitating gene targeting and perhaps for multiplex gene mutagenesis in *C. neoformans* because of the difficulty of gene disruption in basidiomycetous fungi. This work represents the first report in a basidiomycete, and our findings expand the robust CRISPR-Cas9-mediated genome engineering technique to higher fungi.

We have shown the robustness of the CRISPR-Cas9 mutagenesis system in *C. neoformans*. However, we encountered limitations to this technique. For instance, subsequent gene complementation was immediately blocked because of the persistence of gRNA and Cas9 in the mutants. As a bio-tool, CRISPR-Cas9 must be ‘living’ in the host cell to perform its action. In these experiments, the system is normally introduced and expressed in microorganisms and perhaps plants, although the mRNA of Cas9 and gRNA can be injected into mammalian cells. To increase the practical usability of the CRISPR-Cas9 system in *C. neoformans*, we believe that the CRISPR-Cas9 expression cassettes must be eliminated to allow for subsequent manipulation steps, such as complementation. Thus, we attempted to remove these cassettes using several methods. For example, we grew a number of generations of the transformant, although we failed to obtain transformants that lost the CRISPR-Cas9 cassette because the cassettes stably persist within host cells. However, we used a cis construct to demonstrate that the flanking CRISPR-Cas9 DNA beyond the homologous arms was degraded at a high rate (8/18 transformants). The rationale behind the elimination process resembles a counter selection process conducted in several organisms, including human cells and *C. neoformans*, for the purpose of screening recombinants^29^,^30^,^31^,^32^ through negative selection pressure. Obviously, negative selection was not required in our experiments because the recombination frequency was greatly enhanced by CRISPR-Cas9-mediated double-strand breaks (DSB) in *C. neoformans*. The total disruption rate was as high as 80% as calculated by the ratio of the number of colonies to the total transformants. In addition, all eighteen analysed pink transformants had the correct gene disruption for *ADE2* (18/18 HR), and sixteen out of the eighteen randomly selected transformants had the correct gene disruption for *Tsp2-1* (16/18 HR). Importantly, we found that eight out of the eighteen transformants lost the CRISPR-Cas9 system, suggesting that the CRISPR-Cas9 system-mediated double crossover substantially increased the self-destruction of the CRISPR-Cas9 cassette itself (i.e., a ‘suicide’ action for CRISPR-Cas9). We deduced that a temporal cascade of molecular events occurs during this process, whereby the introduced CRIPSR-Cas9 cassette is expressed to generate Cas9 enzyme and gRNA, which in turn cleave the target locus to generate DSB. Subsequently, the double crossover between the genomic copy and the homologous arms on the disruption construct is induced and the flanking gDNA and Cas9 cassettes are excised and released for degradation. In short, our work demonstrates that the CRISPR-Cas9 system can temporarily function during the targeting and elimination process. This suicide concept increases the applicability of the CRISPR-Cas9 system in *C. neoformans* as well as in other organisms and will widen the application of the technique because genetic complementation of the mutated gene is a simple and straightforward method of validating the function-phenotype relationship of the gene. Complementation with a wild-type copy of the targeted gene can exclude potential phenotypic outcomes resulting from secondary mutations caused by off-target editing. Otherwise, resequencing of the entire genome is required in certain circumstances.

Prompt removal of the CRISPR-Cas9 system from the host cells has more significant implications. The persistence of the CRISPR-Cas9 system in the host has the potential to deteriorate off-target mutagenesis and cytotoxicity. A greater number of off-target mutations relative to desired mutations were observed in human cells with the expression approach^11^. Instantaneous loss of CRISPR-Cas9 should minimize off-target mutagenesis and eradicate any potential cytotoxicity of gRNA or Cas9 nuclease. In fact, we found that the expression of Cas9 exhibits a remarkably negative effect on the virulence of *C. neoformans* strain H99 (but not JEC21). Thus, additional caution should be taken when performing virulence tests for mutant strains created by the CRIPSR-Cas9 system. The spontaneous elimination of CRISPR-Cas9 is truly of significance for *C. neoformans*. It is worth noting that the CRIPSR-Cas9 expression cassettes survived in ten of the eighteen analysed transformants, although the anticipated gene disruption was confirmed by two PCR amplifications of the transformants. Considering the rationale of positive-negative selection^29^, we believe that these transformants acquired multiple copies of the linearized plasmid during the electrotransformation. The CRISPR-Cas9 cassettes in the linearized plasmid that underwent HR had been degraded, whereas other linearized plasmids containing CRISPR-Cas9 cassettes might be retained.

Additionally, the spontaneous elimination of CRIPSR-Cas9 may have broader applications in terms of biosafety, which is relevant for several fields, including biomedical applications, such as such as gene therapy, agriculture applications, such as transgenic crops, and food industry applications, such as synthetic microbes. We expect that the concepts detailed in this study will be helpful in the development and application of the CRISPR-Cas9 technique in these fields.

## Materials and Methods

### Microbial strains, media and plasmids

The *C. neoformans* serotype D strain JEC21 is a wild-type strain that is widely used in the field. 4500FOA is a uracil auxotrophic strain of JEC21 that served as the host for the transformation of the CRISPR-Cas9 system. YPD (a complete medium that contains 10% yeast extract, 20% peptone and 20% glucose) was used for the routine growth of *C. neoformans*. The basal medium for selection contained 1.7% YNB (without ammonium and amino acids), 5% ammonium sulphate and 20% glucose. All of the cultures were incubated at 30 °C. All of the vectors that were amplified in *E. coli* DH5α and used for transformation were constructed using the routine plasmid pBluescript II KS(+) (pBS) with either cryptococcal *URA5* or the hygromycin B resistance gene *HygR* as the selection marker. The media were supplemented with 20 mg/L cycloheximide or 100 mg/L hygromycin B as required.

### Reagents, enzymes and primers

T4 DNA ligase and the restriction enzymes used for cloning were obtained from Takara Co. Ltd. (Dalian, China). All quick-cut enzymes were obtained from MBI Fermentas (Shenzhen, China). High-fidelity enzyme polymerases for PCR cloning were obtained from TransGen Biotech (Beijing, China). DNA gel purification kits were obtained from Corning Axygen, China (Jiangsu, China). Sequencing and primer synthesis kits were purchased from GeneWise (Shouzhou, China). For the Southern blot analysis, non-radioactive labelling kits were obtained from Roche-China (Shanghai, China). The primers used in this study are listed in Supplementary Table S2.

### Expression cassettes for Cas9 and gRNA in *C. neoformans*

The 2.0 kb marker *URA5* was amplified by PCR with the primer pair URA5-cassette-F and URA5-cassette-R from the genome of JEC21 and then inserted into the pBS vector between the *Bam*HI and *Pst*I restriction sites, which yielded pBS-URA5 for transformation. Otherwise, PCR fragments carrying the selection marker were directly used to transform 4500FOA. To construct the Cas9 expression cassette, the human codon-optimized Cas9 CDS fragment on pX330 (originally from *Streptococcus pyogenes* SF370, type II CRISPR locus) was obtained from Dr. Zhang Feng (Harvard University, Boston, USA). For the nuclear translocation signals, the SV40 NLS and the nucleoplasmin NLS were retained from pX330 on the sides of the Cas9 CDS. An 800 bp cryptococcal *ACT1* promoter digested with *Xba*I/*Nco*I and a Cas9 coding region with NLSs, followed by a bGHpA terminator with *Nco*I/*Not*I were ligated to the pBS-URA5 vector that was cut with *Xba*I/*Not*I to obtain the pBS-URA5-Pact:Cas9 vector to express Cas9.

To construct the gDNA expression cassette for the production of the single gRNA, we employed the overlap PCR approach. A 20 bp target sequence was used for the synthesis of two complementary primers that were used to fuse the endogenous cryptococcal U6 promoter and the bacterial tracer RNA scaffold (from Dr. F. Zhang); *e.g*., ADE2.B-CnU6-R/ADE2.B-gRNA-F for the gRNA of locus ADE2.B (Table S2). The resulting gDNA cassette contains a U6 promoter, the 20 bp target sequence, the gRNA and a 6 × T terminal signal, in that order. The gDNA cassette digested with *Cla*I/*Xho*I was ligated to the pBS-URA5-Pact:Cas9 vector to obtain the plasmid pBS-URA5-Pact:CRISPR.

### Determination of the CRISPR-Cas9 targets in the genome of *C. neoformans* JEC21

All of the genome sequences were obtained from GenBank. According to several previous reports, the target sequence should be 17–20 nt and the initial transcriptional nuclear acid should be G for the U6 promoter. The 12 nucleotides after the target were named the seed sequence, and they play an important role in the recognition of the Cas9/gRNA complex. Taking all the relevant information into account, the target sites were designed and optimized with the software sgRNAcas9 run in GN_7_S_12_-NGG mode^33^. The software scans putative off-target sites and minimizes the possibility of these sites at the whole genome scale. When a suitable GN_7_S_12_-NGG sequence was not available, then the N_7_S_12_-NGG sequence was used with an additional G at the 5′ terminus.

### Construction of suicide cassettes for the elimination of the CRISPR-Cas9 system

We first assembled the HR structure used to modify the L41-encoding gene. A 1.0 kb L41* fragment containing a mutated leucine codon (TTG) to replace the wild-type CCT codon for proline was created by overlap PCR. The mutant L41* fragment was cloned into pMD19 (a T-vector from TaKaRa) to obtain the donor plasmid pMD-CYH-L41.A. To perform the disruption/replacement of *ADE2*, the upstream fragment of the target site ADE2.C, which was cut with *Kpn*I/*Cla*I, and a downstream fragment, which was digested with *Xba*I/*Bam* HI, were ligated to the pBS-Hyg vector to flank the *HygR* gene and obtain the donor plasmid pBS-Hyg-ADE2.C, and this process was anticipated to occur via double crossover around the locus of ADE2.C.

Similarly, the suicide pBS-Hyg-ADE2.C was cut with *Xba*I/*Not*I, and the gDNA expression cassette was ligated to the side of the HR structure to obtain the plasmid pBS-Hyg-ADE2.C-gDNA to eliminate the gDNA fragment for the following complementation experiment. For the plasmids that can eliminate both the gDNA and Cas9 cassettes, the corresponding homologous fragments flanked *URA5* to obtain the HR structure and the CRISPR-Cas9 cassettes were located to the side of the HR structure. The constructed cassettes were confirmed by restriction mapping and sequencing (GeneWise, Souzhou, China).

### Transformation of *C. neoformans*

Transformation was performed via electroporation according to a previously reported protocol^27^. Approximately 3 μg linear plasmids or PCR products that carried the CRISPR-Cas9 system were purified with a gel purification kit and used to transform the recipient strain 4500FOA. The cells were incubated for over 5 h at 30 °C. For co-transformation, two DNA molecules were mixed in a 1:1 ratio (quantity) and transformed under similar conditions. For example, to edit the L41 gene, 3 μg CRISPR/L41.A and donor DNA were mixed to transform 4500FOA, and 3 μg pBS-URA5-Pact:Cas9 and donor DNA were used as the control. For the ADE2.C target, 3 μg linearized CRISPR/ADE2.C and 3 μg donor DNA were mixed to transform 4500FOA. For the experiment to eliminate gDNA, 3 μg pBS-URA5-Pact:Cas9 and 3 μg gDNA/ADE2.C donor DNA were mixed to co-transform 4500FOA, and the positive colonies were screened on YNB plates with hygromycin and adenine as previously described.

### Reverse PCR to detect the mRNA of Cas9

To confirm the transcription of Cas9 and *URA5* in the transformants, yeast cells were grown in YPD liquid medium at 30 °C for 18 h. Total RNA was prepared following the protocol provided by the RNAiso plus kit (TaKaRa Biotech Co. Ltd., Dalian, China). The first chain of cDNA was synthesized via M-MLV reverse transcriptase (TaKaRa) using a random primer (N_6_). The transcriptional level of Cas9 was determined by PCR amplification with the primer pair Cas9-in-F/Cas9-in-R. The actin-encoding gene *ACT1* mRNA was used as an internal control with the primer pair ACT1-in-F/ACT1-in-R. The primers for *URA5* mRNA detection in the transformants were URA5-in-F/URA5-in-R.

### Southern blot analysis

Yeast strains were cultivated in YPD liquid medium at 30 °C overnight, and the genomic DNA was extracted as previously described^34^. Appropriate restriction enzymes as denoted in the legends were utilized to digest the genomic DNA. The digested DNA was then subjected to separation on 0.7% agarose gels and transferred to N^+^-Magaprobe nylon transfer membranes following the manufacturer’s instructions (Dingguo, Beijing, China). The probe (as indicated in each figure) was labelled with digoxigenin by following the manufacturer’s instructions for the DIG High Primer DNA Labeling and Detection Starter Kit II (Roche China, Shanghai, China).

### Gene complementation

The reconstituted strain was created by introducing a 3.6 kb fragment containing the wild-type copy of *ADE2* into the host strains. The wild-type fragment was PCR amplified from the JEC21 genome using the primer pair ADE2-F/ADE2-R (Table S2). The C3 and E1 strains were electroporated with the 3.6 kb *ADE2* cassette, which was also used as a selectable marker. Transformants were selected on YNB plates and randomly selected for PCR confirmation and sequencing.

## Additional Information

**How to cite this article**: Wang, Y. *et al.* A ‘suicide’ CRISPR-Cas9 system to promote gene deletion and restoration by electroporation in *Cryptococcus neoformans. Sci. Rep.* **6**, 31145; doi: 10.1038/srep31145 (2016).

## Supplementary Material

Supplementary Information
