# Supplementary material for: A ‘suicide’ CRISPR-Cas9 system to promote gene deletion and restoration by electroporation in Cryptococcus neoformans
Source: Sci Rep. 2016 Aug 9;6:31145. doi: 10.1038/srep31145 (PMC4977553; doi:10.1038/srep31145)
Supplement: Supplementary Information [file srep31145-s1.doc]

**Supplementary Information**

**A ‘suicide’ CRISPR-Cas9 system to promote gene deletion and restoration by electroporation in *Cryptococcus neoformans***

Yu Wang^1^, Dongsheng Wei^1^, Xiangyang Zhu^1^, Jiao Pan^1^, Ping Zhang^1^, Liang Huo^2^ & Xudong Zhu^1,2,*^

^1^ National Key Program of Microbiology and Department of Microbiology, College of Life Sciences, Nankai University (DMNU), Tianjin 300071, China

^2^ Beijing Key Laboratory of Genetic Engineering Drug and Biotechnology, Institute of Biochemistry and Biotechnology, College of Life Sciences, Beijing Normal University, Beijing 100875, China

^*^ Correspondence: Xudong Zhu, Institute of Biochemistry and Molecular Biology, College of Life Sciences, Beijing Normal University, Beijing 100875, China

Tel./Fax: +86 10 58804266, 22 23506510

E-mail: zhu11187@bnu.edu.cn

**
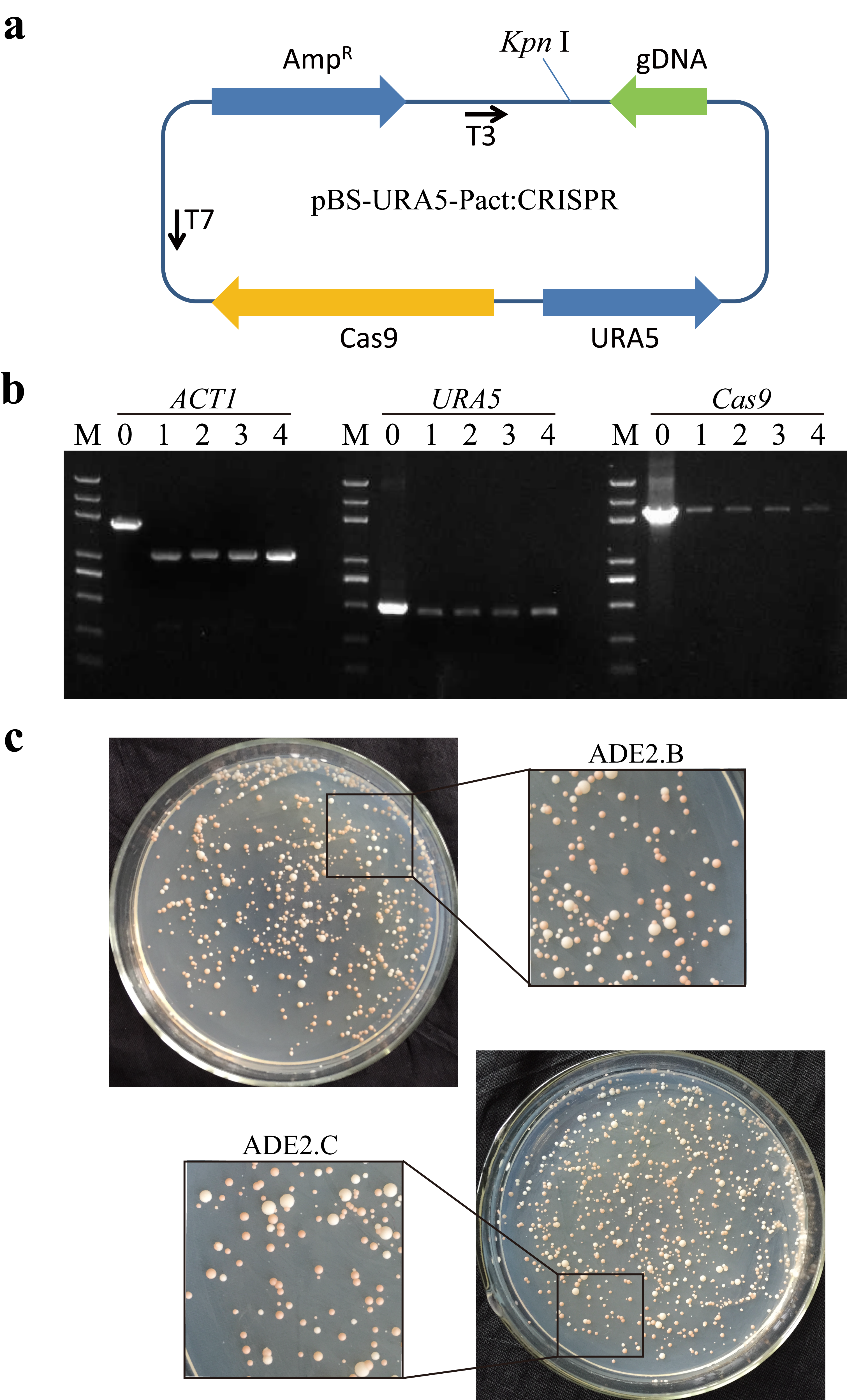
**

**Figure S1**. (**a**) Schematic of the pBS-URA5-Pact:CRISPR vector for gRNA production and Cas9 expression that was constructed based on pBluescript II KS(+). *URA5* is the marker for selection in yeast transformation. The location of the T3/T7 primers was used for sequencing. *Kpn*I was used to linearize the vector. (**b**) Full-length gel results of Figure 1b. M. Trans2K Plus II Marker, 0. Control, genomic DNA as PCR template, 1-4. Total cDNA as PCR template. (**c**) Transformants of CRIPSR-Cas9 (gRNAs are ADE2.B and ADE2.C) into 4500FOA on the YNBA plates (YNB supplemented with 20 mg/L adenine). A large number of *URA5*-positive transformants generated pink colonies.

**
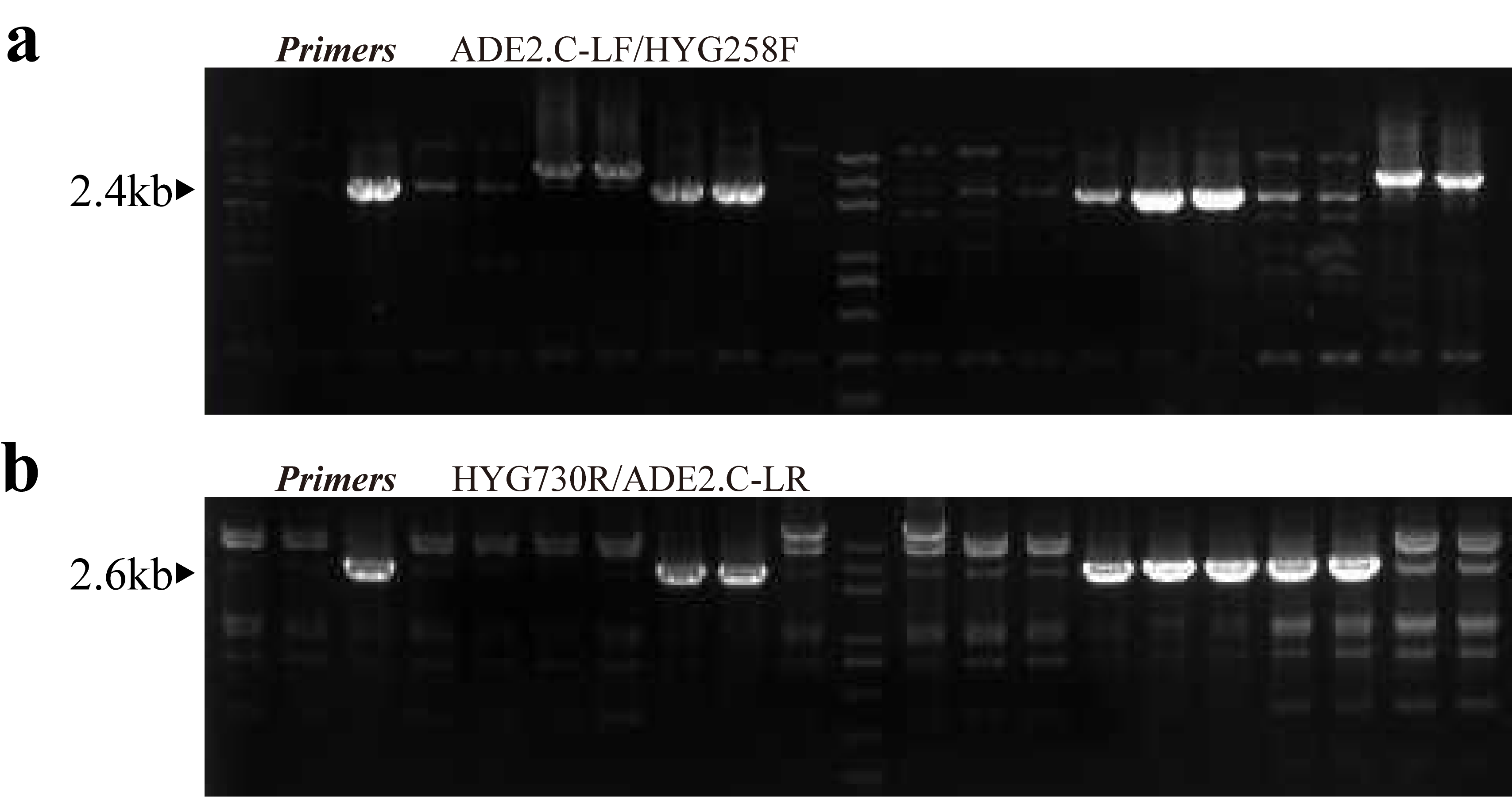
**

**Figure S2**. Full-length gel results of Figure 4b.

**
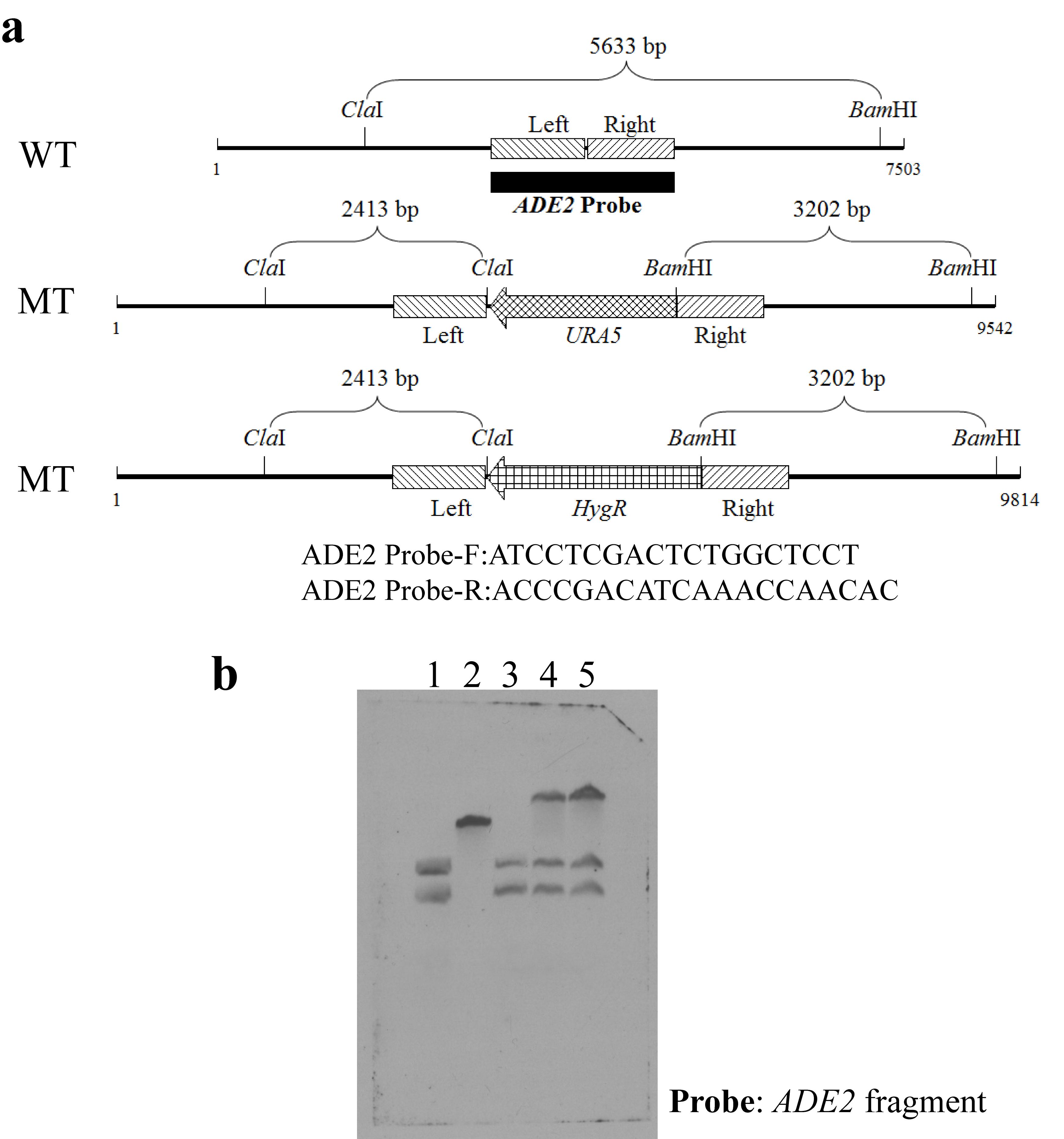
**

**Figure S3**. (**a**) Schematic description for the Southern blotting of the *ADE2* disruptant. Genomic DNA was digested with *Bam*HI and *Cla*I. A 5633 bp fragment was generated by the digestion in the wild-type 4500FOA, whereas two fragments at 3202 bp and 2413 bp were expected for the deletion mutant. The probe was the PCR fragment of *ADE2* amplified with a pair of primers: ADE2 Probe-F/ADE2 Probe-R, which are shown in the solid box. (**b**) Full-length blot results of Figure 4c and Figure 5c. 1. ADE2.C-C3, 2. 4500FOA, 3. ADE2.C-D10, 4. ADE2.C-DC1, and 5. ADE2.C-DC2.


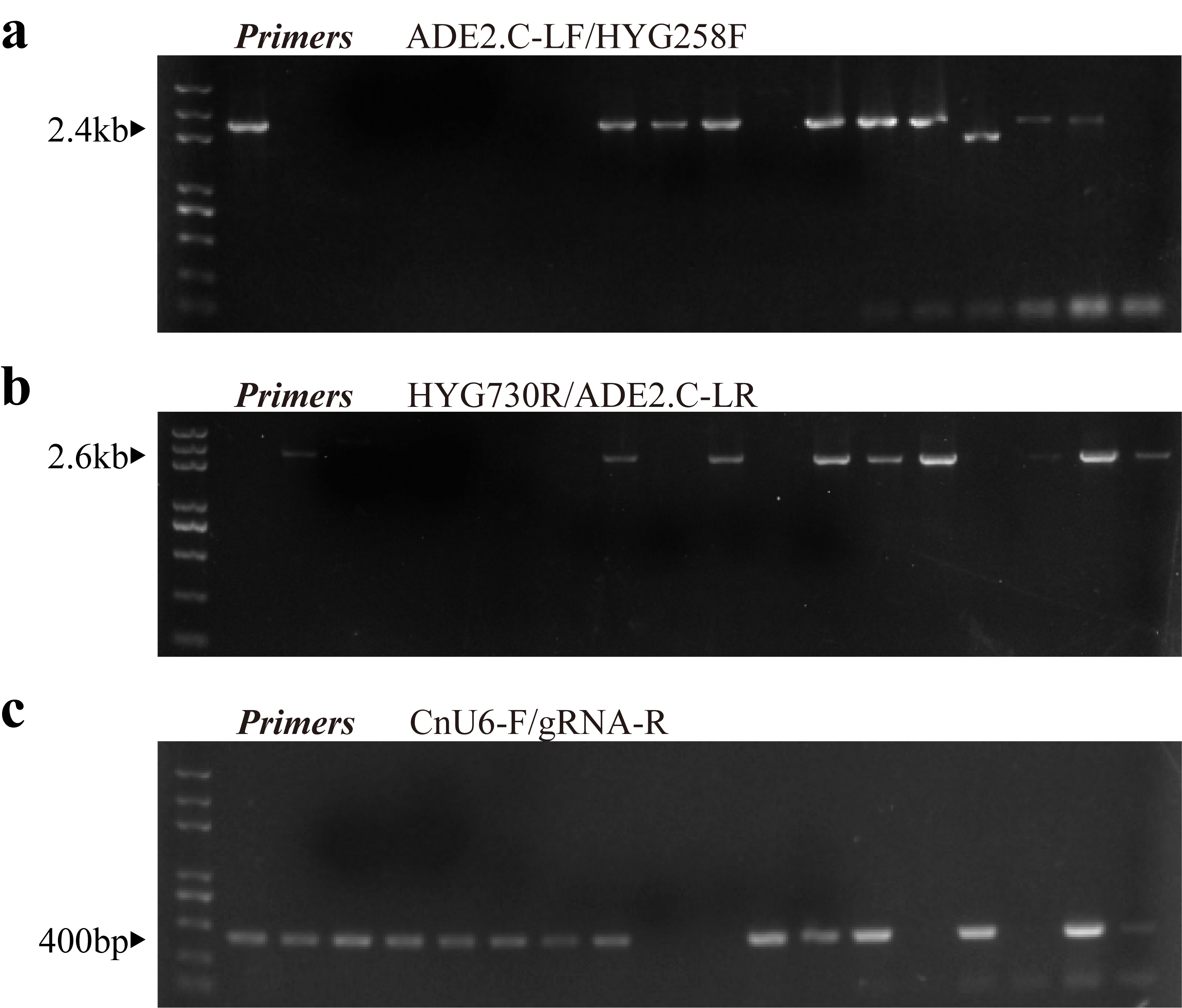


**Figure S4**. Full-length gel results of Figure 5b.

**
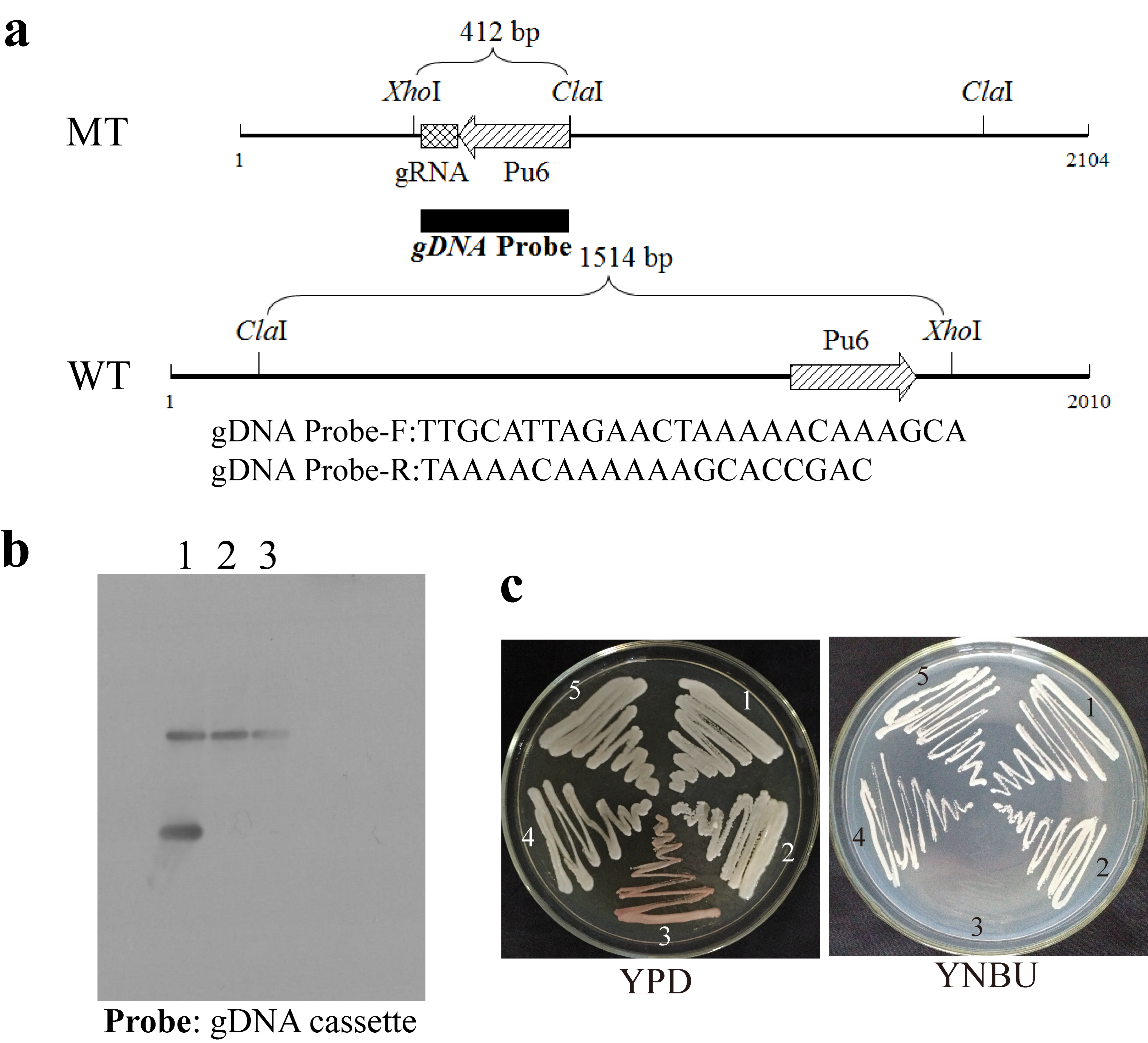
**

**Figure S5**. (**a**) Schematic description of the Southern blotting to show the elimination of gDNA. Genomic DNA was digested with *Cla*I and *Xho*I. A 1514 bp fragment containing the native copy of U6 was present in all of the strains. The 412 bp gDNA band was only present in the strains bearing the gDNA cassette. The probe was the PCR fragment of the gDNA cassette amplified by PCR with a pair of primers: gDNA Probe-F/gDNA Probe-R, shown in the solid box. (**b**) Full-length blot results of Figure 5d. 1. ADE2.C-C3, 2. 4500FOA, and 3. ADE2.C-D10. (**c**) Phenotype for the disruptant D10 and the two complements on YPD and YNB plus uracil (YNBU) at 30°C for 4 days. D10 (No. 3) turned red on YPD and failed to grow on the minimal medium YNBU. The control strains JEC21 (No. 1) and 4500FOA (No. 2) and the complements (No. 4 and 5) remained a light colour on YPD and grew on YNBU.

**
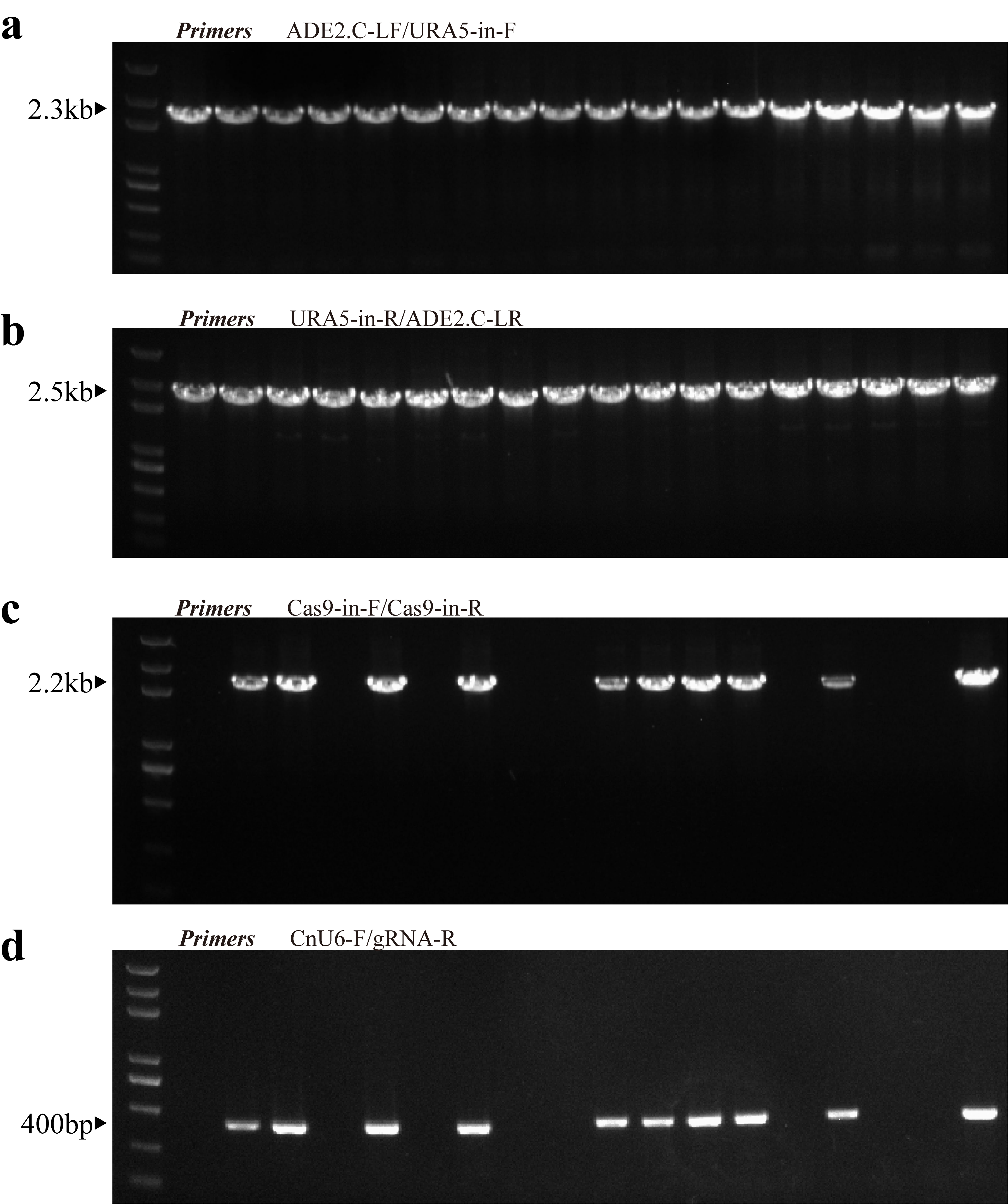
**

**Figure S6**. Full-length gel results of Figure 6b.

**
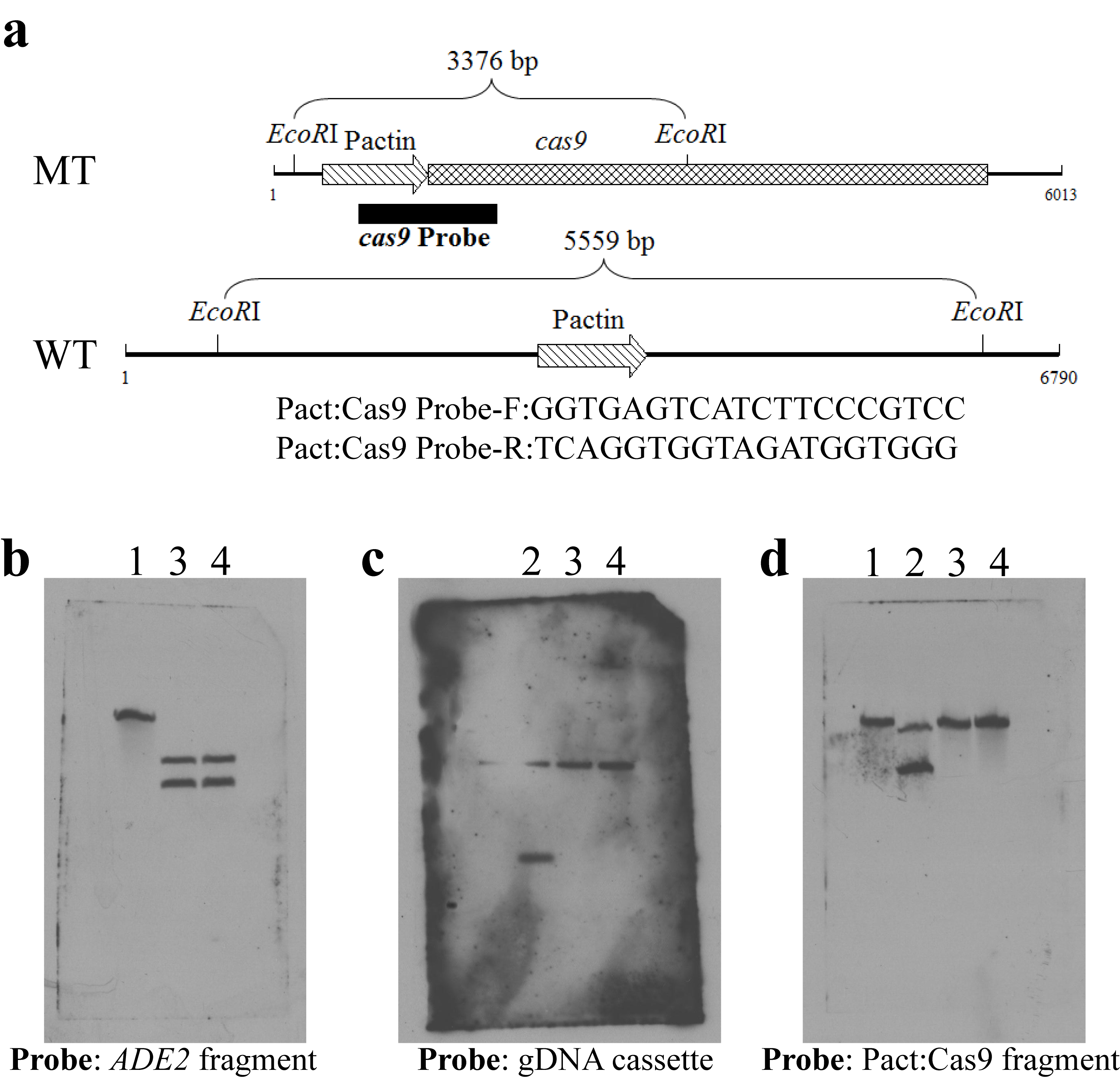
**

**Figure S7**. (**a**) Schematic description of the Southern blotting to show the elimination of Cas9. Genomic DNA was digested with *Eco*RI. A 5559 bp fragment containing the native copy of *ACT1* was present in all of the strains. The 3376 bp Pact:Cas9 band was only present in the strains bearing Cas9 cassette. The probe was the PCR fragment of the Cas9 cassette amplified by PCR with a pair of primers: Pact:Cas9 Probe-F/Pact:Cas9 Probe-R, which are shown in the solid box. (**b-d**) Full-length blot results of Figure 6c-e**.** 1. 4500FOA, 2. ADE2.C-C3, 3. ADE2.C-E1, and 4. ADE2.C-E4.


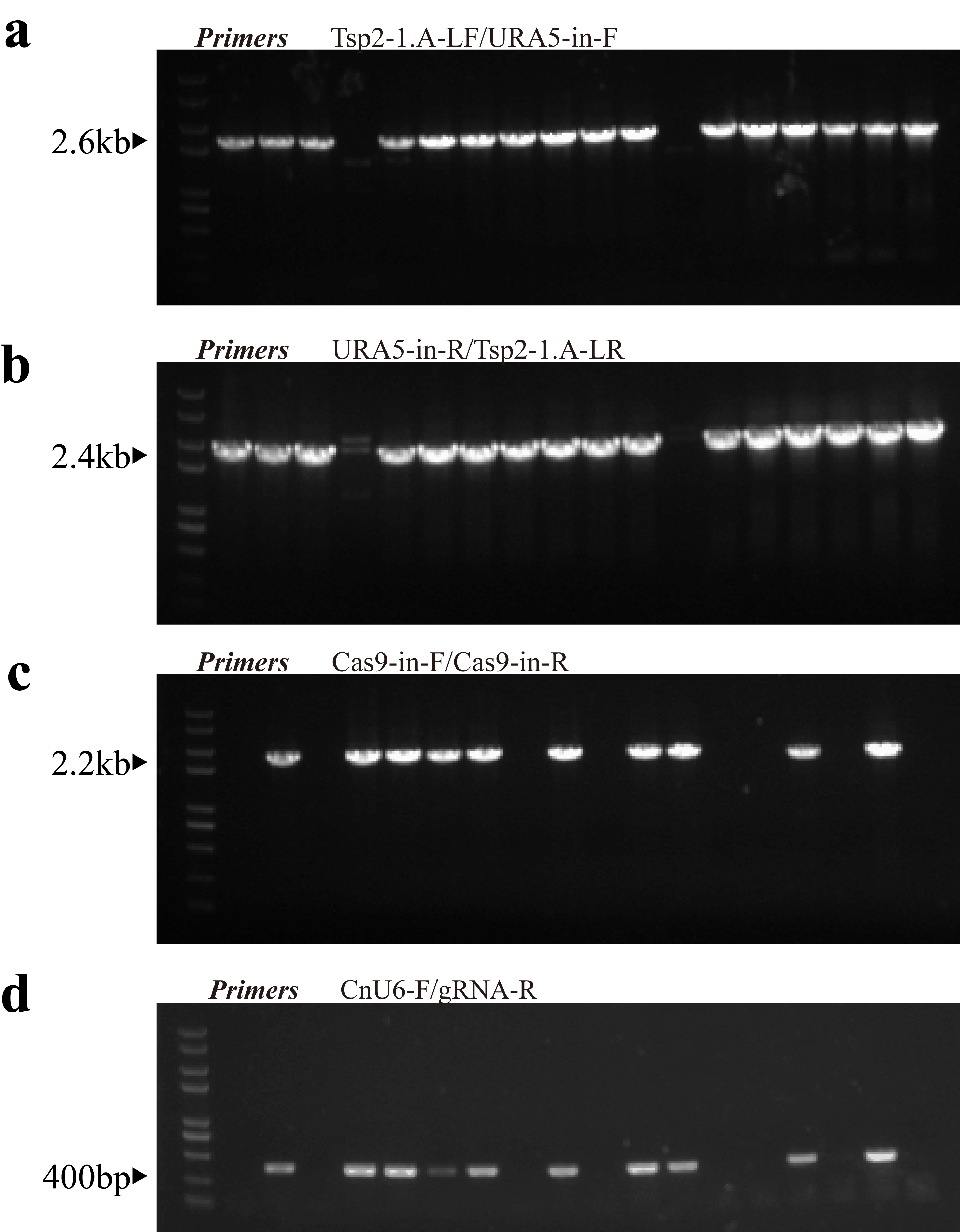


**Figure S8**. Full-length gel results of Figure 7b.

**Table S1**. Sequence of the gDNA cassette.

| **Component** | **Sequence** |
| --- | --- |
| gDNA cassette | TTGCATTAGAACTAAAAACAAAGCATGATTATTACAGTTCATTTATTTTTTAAATTGATCGGCATGCATGCAAAGTATACGTGCAAGGACAATGGTAACCTGCAGGTGTGACCGATAATTATAACCATTTGTTGAGAATGAAGAGGTGAGGAGAAAAACAATGGATGACGGGAAAAAAATAAAAAAACACTGAGACGGCGTGGACCGCCGTCTTATTTGCTTCCGTTATCCGCCAAAGTGGAAATTGCACATACACCGGCAGGGTATACTGTTGNNNNNNNNNNNNNNNNNNNGTTTTAGAGCTAGAAATAGCAAGTTAAAATAAGGCTAGTCCGTTATCAACTTGAAAAAGTGGCACCGAGTCGGTGCTTTTTT |
| CnU6 promoter | TTGCATTAGAACTAAAAACAAAGCATGATTATTACAGTTCATTTATTTTTTAAATTGATCGGCATGCATGCAAAGTATACGTGCAAGGACAATGGTAACCTGCAGGTGTGACCGATAATTATAACCATTTGTTGAGAATGAAGAGGTGAGGAGAAAAACAATGGATGACGGGAAAAAAATAAAAAAACACTGAGACGGCGTGGACCGCCGTCTTATTTGCTTCCGTTATCCGCCAAAGTGGAAATTGCACATACACCGGCAGGGTATACTGTT |
| Target | GNNNNNNNNNNNNNNNNNNN |
| gRNA structure | GTTTTAGAGCTAGAAATAGCAAGTTAAAATAAGGCTAGTCCGTTATCAACTTGAAAAAGTGGCACCGAGTCGGTGC |
| Terminator | TTTTTT |

Table S2. Primers used in this study

| Primers | Sequences |
| --- | --- |
| URA5-cassette-F | CGGGATCCGATCTTGGGGATGGTATTGA |
| URA5-cassette-R | GCAACTGCAGATCCCAGTACTACCCGCTCT |
| ACT1-in-F | ACATCCCCTATACCGCATCC |
| ACT1-in-R | GCTCG GGAGGAGAGACAATC |
| URA5-in-F | CCCTTACTTCTTCAATGCCG |
| URA5-in-R | CACCGAACTCTTCCTCAACC |
| Pactin-F | GCTCTAGAGAGCGGACTCACATAAGCAT |
| Pactin-R | CATGTCCATGGTGGCACCGGTGTTGGGCGAGTTTTACTA |
| Cas9-in-F | CCCACCATCTACCACCTGA |
| Cas9-in-R | GCCGCCAGTAGTTCTTCAT |
| GnU6-F | CCATCGATTTGCATTAGAACTAAAAACAAAGCA |
| gRNA-R | CCGCTCGAGTAAAACAAAAAAGCACCGAC |
| ADE2.B-GnU6-R | TCGTAGGCAAGGGTCTTAGCAACAGTATACCCTGCCGGTG |
| ADE2.B-gRNA-F | GCTAAGACCCTTGCCTACGAGTTTTAGAGCTAGAAATAGCAAGTT |
| ADE2.C-GnU6-R | TCTTACGGGCCTTTCGGCTCAACAGTATACCCTGCCGGTG |
| ADE2.C-gRNA-F | GAGCCGAAAGGCCCGTAAGAGTTTTAGAGCTAGAAATAGCAAGTT |
| L41.A-gRNA-R | AGCCTGTCTTCCACAAGAACAACAGTATACCCTGCCGGTG |
| L41.A-GnU6-F | GTTCTTGTGGAAGACAGGCTGTTTTAGAGCTAGAAATAGCAAGTT |
| Tsp2-1.A-gRNA-R | AAGCGGGAGCGCCTGTGAGCAACAGTATACCCTGCCGGTG |
| Tsp2-1.A-GnU6-F | GCTCACAGGCGCTCCCGCTTGTTTTAGAGCTAGAAATAGCAAGTT |
| L41.A-up-F | GAGATTATGTGCGTTACGCTGTT |
| L41.A-up-R | CAACTTGGTCTGACCACCGTAAC |
| L41.A-down-F | GTTACGGTGGTCAGACCAAGTTGGTCTTCCACAAGAAGGCTAA |
| L41.A-down-R | AATTGAATGATGCTACCCCAAG |
| L41.A-up-LF | ATCCCTCAAGAGTACTGCTCAGC |
| ADE2.C-up-F | GGGGTACCATCCTCGACTCTGGCTCCT |
| ADE2.C-up-R | CCATCGATCTCAGCTTTGCCGTAGAGA |
| ADE2.C-down-F | CGGGATCCACATCACGGTGACTGCCGA |
| ADE2.C-down-R | GCTCTAGAACCCGACATCAAACCAACAC |
| ADE2.C-LF | CAGCAGCTTCAGTCATGCA |
| ADE2.C-LR | TGTCGATGGCAATCGTGTC |
| ADE2-F | CCTGCACATACTCCATGACA |
| ADE2-R | TGAAGAAGAAGAGCAGGAGG |
| Hyg258F | CCTGACCTATTGCATCTCCC |
| Hyg730R | GCTCCATACAAGCCAACCAC |
| Tsp2-1.A-up-F | CCATCGATTCCGGGATGGAAGAAGTGGAA |
| Tsp2-1.A-up-R | CCATCGATCTGAGTGTCGGATGGCATACC |
| Tsp2-1.A-down-F | CGGGATCCCTTCGCATCCAAATTCGCTC |
| Tsp2-1.A-down-R | CGACCCTCTGTTCCAAGGTA |
| Tsp2-1.A-LF | TTGGTGCAGGGGATGGTCTT |
| Tsp2-1.A-LR | ACCACAGCATCGCAACTGGA |
| ADE2-Probe-F | ATCCTCGACTCTGGCTCCT |
| ADE2-Probe-R | ACCCGACATCAAACCAACAC |
| gDNA Probe F | TTGCATTAGAACTAAAAACAAAGCA |
| gDNA Probe R | TAAAACAAAAAAGCACCGAC |
| Pact:Cas9 Probe-F | GGTGAGTCATCTTCCCGTCC |
| Pact:Cas9 Probe-R | TCAGGTGGTAGATGGTGGG |
